# Using Click-Chemistry for Visualizing *in Situ* Changes of Translational Activity in Planktonic Marine Bacteria

**DOI:** 10.3389/fmicb.2017.02360

**Published:** 2017-12-01

**Authors:** Ainara Leizeaga, Margarita Estrany, Irene Forn, Marta Sebastián

**Affiliations:** Departament de Biologia Marina i Oceanografia, Institut de Ciències del Mar, Consejo Superior de Investigaciones Científicas (CSIC), Barcelona, Spain

**Keywords:** BONCAT, marine bacteria, click-chemistry, marine ecology, single-cell microbiology

## Abstract

A major challenge in microbial ecology is linking diversity and function to determine which microbes are actively contributing to processes occurring *in situ*. Bioorthogonal non-canonical amino acid tagging (BONCAT) is a promising technique for detecting and quantifying translationally active bacteria in the environment. This technique consists of incubating a bacterial sample with an analog of methionine and using click-chemistry to identify the cells that have incorporated the substrate. Here, we established an optimized protocol for the visualization of protein-synthesizing cells in oligotrophic waters that can be coupled with taxonomic identification using Catalyzed Reporter Deposition Fluorescent *in Situ* Hybridization. We also evaluated the use of this technique to track shifts in translational activity by comparing it with leucine incorporation, and used it to monitor temporal changes in both cultures and natural samples. Finally, we determined the optimal concentration and incubation time for substrate incorporation during BONCAT incubations at an oligotrophic site. Our results demonstrate that BONCAT is a fast and powerful semi-quantitative approach to explore the physiological status of marine bacteria.

## Introduction

Microbial activity and its response to environmental factors significantly influence oceanic biogeochemical cycles (Azam, 1998; Falkowski et al., 2008). Although the activity of marine bacteria has been traditionally estimated through bulk measurements, single-cell techniques have revealed significant heterogeneity in the metabolic state of individual cells within populations (Cottrell and Kirchman, 2003; Alonso-Sáez and Gasol, 2007; Del Giorgio and Gasol, 2008). Those findings have pointed out the importance of using single-cell techniques to identify microbes that are actively contributing to global biochemical cycles.

The activity of individual cells and bacterial growth have been studied primarily by methods that detect protein or nucleic acid synthesis. ^3^H-based microautoradiography (Fuhrman and Azam, 1982; Smith and Azam, 1992; Cottingham et al., 2005) has been the most widely used, but it requires specialized laboratory facilities and time-intensive sample processing. A non-radioisotopic, fluorescence-based method detecting incorporation of the thymidine analog bromodeoxyuridine 68 (BrdU) has been used as an alternative (Pernthaler et al., 2002b; Hamasaki et al., 2004), with promising results. However, this method is tedious and involves multiple steps during sample processing that can lead to partial sample loss. Secondary ion mass spectrometry of single cells (nanoSIMS) has also been used to quantify single-cell activities (Musat et al., 2008; Arandia-Gorostidi et al., 2017), and it provides high spatial resolution combined with sensitive quantification of stable isotope-labeled compounds. The downside, however, is the relatively low-throughput and the need for very expensive instrumentation, limiting its usage in the field.

A new and promising approach to investigate single cell activity, based on biorthogonal non-canonical compounds coupled with click-chemistry, has been developed over the last decade (Beatty et al., 2005; Dieterich et al., 2006). This technique uses synthetic amino acids (analogs for methionine) that do not interfere with cell processes and takes advantage of the substrate promiscuity of the translational machinery (Kiick et al., 2002). Upon incorporation of the synthetic amino acid into newly synthesized proteins, translationally active cells can be detected fluorescently via azide-alkyne click-chemistry and fluorescence microcopy. The method, referred to as bioorthogonal non-canonical amino acid tagging (BONCAT; Dieterich et al., 2006), has been applied successfully to identifying protein-synthesizing microbes either in culture or natural samples (Hatzenpichler et al., 2014, 2016; Samo et al., 2014). Hatzenpichler et al. (2014) pioneered the use of BONCAT with complex environmental samples, such as oral biofilms, freshwater, and anoxic sediments. They also demonstrated that BONCAT can be combined with Fluorescence *in Situ* Hybridization to link microbial identity with translational activity. Samo et al. (2014) applied BONCAT to marine systems and performed a suite of comprehensive experiments comparing BONCAT results with microautoradiography and bulk radioisotope incorporation to estimate protein-synthesis rates. These studies set the stage for the application of BONCAT to environmental samples, but methodological discrepancies between the two studies warrant the development of a standardized protocol. For example, Samo et al. (2014) used a coverslip to help maintain the reducing conditions necessary for the click-reaction, whereas Hatzenpichler et al. (2014) did not. Furthermore, Samo et al. (2014) introduced a filter-transfer-freeze (FTF) step to reduce the background signal but this step may result in loss of cells and hamper downstream processing of the samples.

The aim of this study was to establish an optimized BONCAT protocol that can be applied in combination with Catalyzed Reporter Deposition Fluorescent *in Situ* Hybridization (CARDFISH) for the study of planktonic bacteria. CARDFISH uses horseradish peroxidase (HRP)-labeled oligonucleotide probes and tyramide signal amplification, and was developed as an improvement of FISH to allow detection of cells with low ribosomal content that are often prevalent in oligotrophic waters. A critical step for CARDFISH is the permeabilization of the cells prior to diffusion and hybridization of the probe (Pernthaler et al., 2002a). BONCAT has been successfully combined with CARDFISH to assess the activity of microbes in deep sea sediments (Hatzenpichler et al., 2016). However, in that study the permeabilization step involved the use of HCl and SDS, which differs from the most widely used protocols that employ a treatment with lysozyme and achromopeptidase (Sekar et al., 2003).

We first evaluated the benefit to the BONCAT signal of using a coverslip during the click-reaction. Next, we examined the effect of permeabilizing cells in both cultures and environmental samples on BONCAT reactions. Then we applied BONCAT to monitor growth in cultures of marine bacteria to demonstrate the utility of this technique for visualizing changes in the translational activity of single cells. We determined the optimal concentration of substrate and incubation duration for studies in oligotrophic waters. We confirmed that BONCAT signal intensity correlated with protein synthesis rates and provided evidence of the power of this technique to explore changes in single cell activity with natural samples. Finally, we demonstrated that BONCAT can be successfully coupled with CARDFISH to link activity and identity of planktonic prokaryotes.

## Materials and Methods

### BONCAT Protocol

The BONCAT protocol consists of two phases, the first being the incubation of the samples with the methionine analog (L*-*homopropargylglycine, HPG), fixation, and immobilization of the cells onto a filter membrane. The second phase is the click-reaction, in which the alkyne terminal group of the HPG binds covalently to the azide terminal of a chosen fluorochrome. A standard BONCAT protocol was designed based on Hatzenpichler and Orphan (2015) with slight modifications introduced by Samo et al. (2014), combining them with steps used in standard CARDFISH protocols.

#### Incubation with the Substrate L-Homopropargylglycine (HPG)

L-Homopropargylglycine (1067-25 ^1^) was dissolved in dimethyl sulfoxide (DMSO) to yield a 19.7 mM substrate solution and stored at -20°C. Working solutions (200 μM) were prepared in filtered-sterilized milliQ water and kept at 4°C in the dark. Seawater samples were incubated with varying concentrations of HPG (20–1000 nM) for a certain time (0.5–4 h) at room temperature (RT) in the dark. Samples were then fixed with 0.2 μm-filtered paraformaldehyde (PFA, final concentration 1% [v/v]) overnight at 4°C. The samples were then gently filtered through a 0.2-μm pore size polycarbonate filter, which was placed on top of a 0.8-μm cellulose acetate filter, washed three times with sterile milliQ water, and frozen at -80°C until further processing. After thawing, the filters were dipped in 0.1% low-gelling-point agarose, dried at 37°C, and then dehydrated with 95% ethanol (EtOH). This allowed attachment of the cells to the filters to prevent cell loss during permeabilization and downstream procedures. Cell walls were permeabilized with lysozyme (10 mg ml^-1^; 0.05 M EDTA, 0.1 M Tris–HCl, 1 h) and achromopeptidase (60 U ml^-1^, 0.01 M NaCl, 0.01 M Tris–HCl, pH 7.6, 30 min) at 37°C following standard protocols (Sekar et al., 2003), unless noted in the text. Each filter was cut into either 1/10 or 1/6 slices using a sterile razor blade. The remaining portion was stored (-80°C). Cu(I)-catalyzed click-chemistry was later performed, following Hatzenpichler and Orphan (2015).

#### Click-Reaction

Two different azide dyes were used for the dye premix during this study: Cy3 azide (green excitation, orange emission) and carboxyrhodamine azide (Cr110; blue excitation, green emission). Stocks (1 mM final concentration) were prepared in DMSO for Cy3 and dimethylformamide for CR110.

For the click-reaction, a dye-premix was prepared by mixing 1.25 μl of filter-sterilized 20 mM CuSO_4_, 2.5 μl of filter-sterilized 50 mM Tris[(1-hydroxypropyl-1H-1,2,3-triazol-4-yl)methyl]amine (THPTA ^2^), and 1 μl of the azide dye (in our case either Cy3 or CR110, 5 μM final concentration). This premix was allowed to react for 3 min at RT in the dark. In the meantime, 12.5 μl of a freshly prepared 100 mM solution of sodium ascorbate and 12.5 μl of a freshly prepared 100 mM solution of aminoguanidine hydrochloride were added to 221 μl of phosphate-buffered saline (PBS). Then, for the click-reaction mix, the dye-premix was added to the PBS–ascorbate–aminoguanidine mix and the tube was inverted once. Then the pieces of filters were placed on slides and each piece covered with 20 μl of the click-reaction mix and incubated in the dark at RT in wet chambers for 30 min. Unless otherwise stated in the text a coverslip was used to cover the filters to minimize exposure to oxygenated conditions. After the click-reaction, filters were washed three times for 3 min each in PBS-filled petri dishes and finally they were dehydrated by incubating them for 3 min in increasing concentrations of EtOH: 50, 70, and 96%, at RT. Filters were counterstained with 4′,6-diamidino-2-phenylindole (DAPI, 10 μg ml^-1^ final concentration) and analyzed through epifluorescence microscopy.

Killed controls (samples fixed before the HPG addition) were included with all sets of samples to correct for background fluorescence from naturally occurring azides and check for community shifts (this is particularly important when long incubations are performed).

### Bacterial Cultures

Two bacterial strains were used in this study: the flavobacterium *Dokdonia* sp. MED134, and the alphaproteobacterium *Phaeobacter* sp. MED193. For the experiments, cultures were grown at RT in 250 ml autoclaved marine seawater supplemented with 0.3 μM of K_2_HPO_4_, 2 μM of NH_4_Cl, and 20 μM of glucose.

### Single Cell Activity during Growth of a Bacterial Culture

We followed the changes in single-cell activity of the alphaproteobacterium *Phaeobacter* sp. MED193, which belongs to the ubiquitous *Roseobacter* clade, over time. *Phaeobacter* sp. MED193 was inoculated into duplicate 250 ml Nalgene bottles with sterile marine water to start the culture at 5 × 10^4^ cells ml^-1^. Six time-points were sampled (0, 17, 23, 44, 68, and 160 h after inoculation). Incubations were done with 20 nM of HPG final concentration in 10-ml duplicate samples, during 2 h. Incubations were terminated by the addition of PFA and, within 24 h, filtered onto a 0.2-μm polycarbonate filter. The optimized BONCAT protocol was performed as described above using a coverslip for the click-reaction. Cy3-azide was used for the dye premix and all filters were incubated with the same reaction mix. Microscopic analyses were performed using an Olympus DP72 camera (Olympus America Inc.) connected to a Olympus Bx61 epifluorescence microscope at 1000× magnification. The following fluorescence filters were used for DAPI: excitation BP330-385, dichroic mirror DM400, and barrier filter BA420, and for Cy3: excitation BP510-550, dichroic mirror DM570, and barrier filter BA591.

Images were acquired using the camera through the CellF software and analyzed using the automated image analysis software ACMEtool ^3^. All images (at least 10 fields/filter) were acquired using 20 ms exposure time for DAPI and 250 ms exposure time for BONCAT (Cy3).

The percentage of translationally active cells (BONCAT+ cells) was calculated in relation to the DAPI counts. The intensity of the BONCAT+ cells was assessed using the mean gray value, which is the sum of the gray values of all the pixels in the cell divided by the number of pixels. The intensities were rank-ordered to obtain the maximum and minimum values and the intensity range was then equally divided into three groups: high intensity (top third), intermediate intensity (middle third), and low intensity cells (bottom third). Finally, the percentage of each group within the BONCAT+ cells was calculated at each time point.

### Relationship between Bacterial Activity and BONCAT

We compared BONCAT with bulk bacterial protein synthesis rates (measured as ^3^H-leucine incorporation) to confirm that the BONCAT signal was a good proxy for activity. These tests were performed using samples from surface waters collected at the Blanes Bay Microbial Observatory (BBMO), an oligotrophic coastal station located in the North-Western Mediterranean Sea (Gasol et al., 2012), and a mesocosm experiment that covered a wider range of trophic conditions. Samples from the BBMO were incubated for BONCAT and ^3^H-leucine incorporation for 1, 2, 3, 4, and 5 h. In the case of the mesocosms experiment, samples for BONCAT and ^3^H-leucine incorporation were taken at several time points along 30 h, but the incubation always lasted 0.5 h.

Bacterial protein synthesis was estimated from the uptake of ^3^H-leucine using the centrifugation procedure (Kirchman et al., 1985). Four replicates of 1.2 ml and two trichloroacetic acid (TCA)-killed controls were incubated with ^3^H-leucine at a final concentration of 40 nM. Incubations were performed in the dark at *in situ* temperature for 1.5 h and stopped with 5% TCA, final concentration. The samples were kept frozen at -20°C until processing following Smith and Azam (1992).

### Using BONCAT to Assess Changes in Activity in Natural Samples

#### BONCAT in Growth Arrested Cells

To demonstrate that HPG is specifically incorporated only in protein-synthesizing cells, we performed BONCAT on cells that were treated with 0.1 mg/ml of serine hydroxamate (SHX). SHX is a serine analog that stops cellular growth by competitively inhibiting seryl-tRNA synthetase (Tosa and Pizer, 1971). It has been widely used with *Escherichia coli* and other clinical bacterial strains to simulate amino acid starvation and study the growth arrest response (Nguyen et al., 2011). Tests were performed using 70 ml flasks with water from the Barcelona coast. Samples were taken at 0, 0.5, 1, and 2 h after addition of SHX. A treatment without SHX was also sampled as control. A killed control was included with every set of samples.

#### Changes in Activity during Manipulation Experiments

We performed a manipulation experiment to test the use of BONCAT in addressing changes in the activity of individual cells in response to different treatments. Water was collected from the Barcelona beach using a 20 μm mesh to sieve out microphytoplankton and microzooplankton. Four different treatments were tested: control, glucose addition (30 μM glucose), increasing temperature to 35°C, and a predator-free treatment (predators were removed by filtering through a 1 μm-pore size filter). All treatments were incubated in 275 ml flasks in the dark for 1 day at RT except for the temperature treatment which was incubated in a temperature controlled chamber. The different treatments were sampled at 0, 2, 6, and 24 h after the experiment started. Nine milliliters of samples was incubated with final concentrations of 20 nM HPG for 1 h and 1 ml of PFA was used for fixing the samples. The optimized BONCAT method was performed using the fluorochrome Cr110-azide for the dye premix.

Images were acquired using an Axio Imager.Z2m epifluorescence microscope connected to a Zeiss camera (AxioCam MRm, Carl Zeiss MicroImaging, S.L., Barcelona, Spain) at 630× magnification through the Axiovision software, and analyzed using ACMEtool. The following fluorescence filters were used for DAPI: 370/40 nm excitation, 425/46 BP emission, and FT 395 beam splitter, and for CR110: 475/30 excitation, 527/54 BP emission, and FT 495 beam splitter. All images (between 15 and 35 images per filter) were acquired using 20 ms exposure time for DAPI and 500 ms exposure time for BONCAT (CR110). The percentage of BONCAT+ cells was calculated in relation to the DAPI counts. ACMEtool was used for subtracting the background of the samples. For that purpose, the signal-to-background ratio of the cells was adjusted until the killed control displayed 0–1.5% of BONCAT+ cells because of background noise observed from DAPI staining of the killed controls. BONCAT-positive cell intensities were analyzed as described above. The percentage of each intensity group was calculated for each treatment at each time point.

### Optimizing Substrate Concentration and Incubation Time for Oligotrophic Waters

In order to understand the time and concentration dependence of HPG incorporation in oligotrophic waters, we performed incubations with seawater from the BBMO using 100, 500, and 1000 nM of HPG and two different incubations times (1 and 4 h) for each concentration. These tests were carried out monthly over June–September 2016.

### Coupling BONCAT with CARDFISH

Cells were attached and permeabilized as described above and filters were cut into multiple pieces and hybridized with one of the following HRP-labeled probes: GAM42a with its unlabeled competitor probe to target Gammaproteobacteria (Manz et al., 1992), Alf968 to target Alphaproteobacteria (Neef, 1997), and SAR11-441R to target the Alphaproteobacteria SAR11 (Morris et al., 2002), following the protocol described in Pernthaler et al., (2004). Specific hybridization conditions were established by addition of formamide to the hybridization buffers (45% formamide for the SAR11 probe and 55% for the other probes). Hybridization was performed overnight at 35°C. For amplification, we used tyramide labeled with Alexa 594. The click-reaction for BONCAT analyses was subsequently performed using the azide dye CR110. We counterstained CARDFISH preparations with DAPI (final concentration 10 μg ml^-1^). DAPI, BONCAT, and CARDFISH-stained cells were visualized by epifluorescence microscopy as described above.

### Data Analysis

Data treatment and statistical analyses were performed with the R Statistical Software (R Core Team, 2016) using version 3.3.2. The manipulation experiment data were analyzed with a mixed-model (sampling times as within-subject factor and treatments as fixed between-subject factors) using the *nlme* package (Pinheiro et al., 2017).

## Results

### Protocol Optimization

#### Optimization of the Click Reaction

**FIGURE 1:**
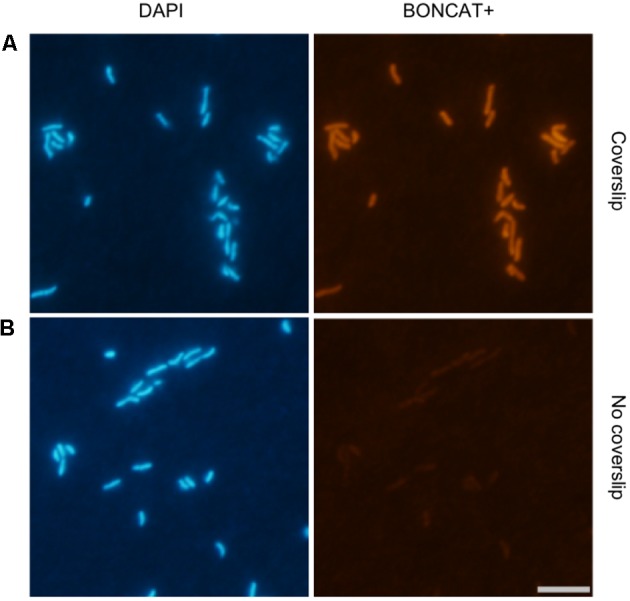
Reducing conditions during the click reaction are crucial to enhance the BONCAT signal. Micrographs show DAPI and BONCAT+ cells of the flavobacterium *Dokdonia* sp. MED134 using the fluorochrome Cy3 azide: **(A)** a coverslip was used during the click-reaction to minimize the exposure of the samples to oxygen, **(B)** no coverslip was used. Scale bar for all images = 5 μm.

In previous studies (Samo et al., 2014; Hatzenpichler and Orphan, 2015), the protocol for the Cu(I)-catalyzed click-reaction was slightly different. Reducing conditions are needed to keep the metal in its Cu(I) oxidation state. Because of the instability of Cu(I) under oxygenated conditions, Cu is added in excess and the reaction is performed in the presence of the reductant sodium ascorbate (Hatzenpichler and Orphan, 2015). Samo et al. (2014) used a coverslip to minimize oxygen exchange during the click-reaction, while Hatzenpichler and Orphan (2015) did not. In order to verify that the coverslip improved the detection of protein synthesizing cells, we performed BONCAT with a culture of the flavobacterium *Dokdonia* sp. MED134, testing the effect of the coverslip in the reaction. Our results showed a remarkable signal improvement when the coverslip was used (**Figure 1**), as observed by Samo et al. (2014).

#### Effect of Permeabilization of the Cells in the BONCAT Signal

**FIGURE 2:**
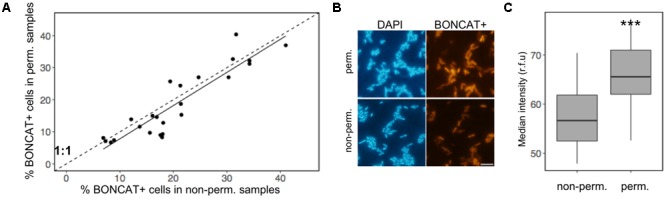
Effect of permeabilization in the BONCAT signal: **(A)** relationship between the percentage of BONCAT+ cells in permeabilized and non-permeabilized cells from environmental samples, **(B)** micrographs of DAPI-stained cells and BONCAT+ cells of a culture of the alphaproteobacterium *Phaeobacter* sp. MED193 with and without permeabilization treatment, **(C)** box plot of the median intensity of the fluorescence of non-permeabilized and permeabilized cells in environmental samples. The lower and upper hinges correspond to the first and third quartiles (the 25th and 75th percentiles), the solid line represents the median of the values, the whiskers represent 1.5 ^∗^ (the distance between the first and third quartiles). Asterisks denote statistical significance (ANOVA test, *p* = 0.0001). The fluorochrome Cy3 azide was used for the BONCAT assay.

The percentage of BONCAT+ cells was very similar before and after the permeabilization treatment (**Figure 2A**). However, the fluorescence intensity of the BONCAT+ cells was significantly higher after permeabilization in experiments with both bacterial cultures and environmental samples (**Figures 2B,C**).

### Single Cell Activity during Growth of a Bacterial Culture

**FIGURE 3:**
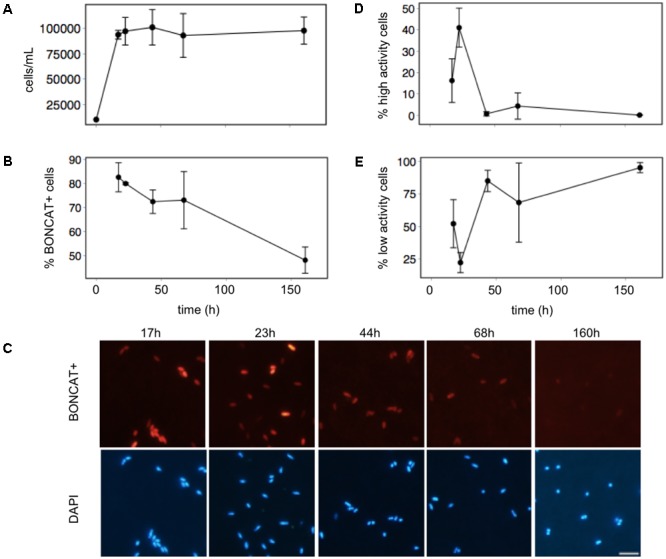
Using BONCAT to monitor changes in the translational activity of cells during the growth curve of the bacterium *Phaeobacter* sp. MED 193: **(A)** total number of cells, **(B)** percentage of translationally active cells, **(C)** micrographs of BONCAT+ cells and total cells (DAPI-stained) during the growth curve. Scale bar for all images = 5 μm, **(D)** percentage of high activity cells (top-third of fluorescence intensity, see section “Materials and Methods” for details), **(E)** percentage of low activity cells (bottom-third of fluorescence intensity). The fluorochrome Cy3 azide was used for the BONCAT assay.

A rapid increase of the total number of cells (visualized with the nucleic acid stain DAPI) was observed within the first 17 h (**Figure 3A**), after which stationary phase was reached. In contrast, the number of translationally active cells (hereafter described as “active” cells) decreased along the growth curve (**Figure 3B**), and after 160 h only 48% of the cells were active.

In addition to the decrease in the percentage of active cells, there were remarkable changes in the fluorescence intensity of the cells (**Figure 3C**). Cells with high translational activity (top third of the fluorescence intensity range) were more abundant in the first two time-points, displaying a maximum 23 h after inoculation (**Figure 3D**). The activity of the cells then decreased, as visualized by a much less intense BONCAT signal in the micrographs (**Figure 3C**). The pool of active cells at the end of the experiment was entirely composed by low activity cells (bottom third of fluorescence intensity, **Figure 3E**).

### Relationship between Bulk Bacterial Protein Synthesis and BONCAT+ Cells in Natural Samples

**FIGURE 4:**
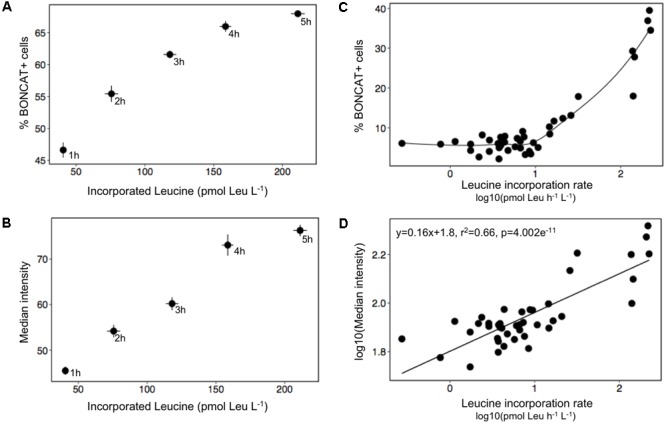
Relationship between prokaryotic protein synthesis and BONCAT results taking into account percentage of BONCAT+ cells (upper panel) and the median intensity of the fluorescent BONCAT signal (lower panel). **(A,B)** Values obtained from an environmental sample taken at the oligotrophic BBMO using different incubations times (1, 2, 3, 4, and 5 h). Leucine incorporated values represent total incorporation within each incubation time. **(C,D)** Data obtained during a mesocosm experiment with natural waters. In this case, BONCAT results are compared with leucine incorporation rates.

The percentage of BONCAT+ cells highly correlated with the leucine incorporated over the first 4 h of incubation in surface waters collected at the BBMO, and after that the percentage of BONCAT+ cells reached saturation (**Figure 4A**). The median fluorescence intensity of the cells was also proportional to the leucine incorporated during the first 4 h (**Figure 4B**).

In the mesocosm experiment, that covered a wide range of trophic conditions, we found that the percentage of BONCAT positive cells highly correlated with bacterial protein synthesis rates, but only above a certain threshold (∼10 pmol Leu l^-1^ h^-1^, **Figure 4C**). However, when the median intensity of the fluorescence of BONCAT+ cells was used instead of percentage of active cells, the correlation with protein synthesis rates was much higher (**Figure 4D**), providing further proof that BONCAT is a suitable technique for visualizing bacterial activity.

### Using BONCAT for Measuring Changes in Single-Cell Activity of Natural Samples

#### BONCAT Signal Is Not Detected in Growth Arrested Cells

**FIGURE 5:**
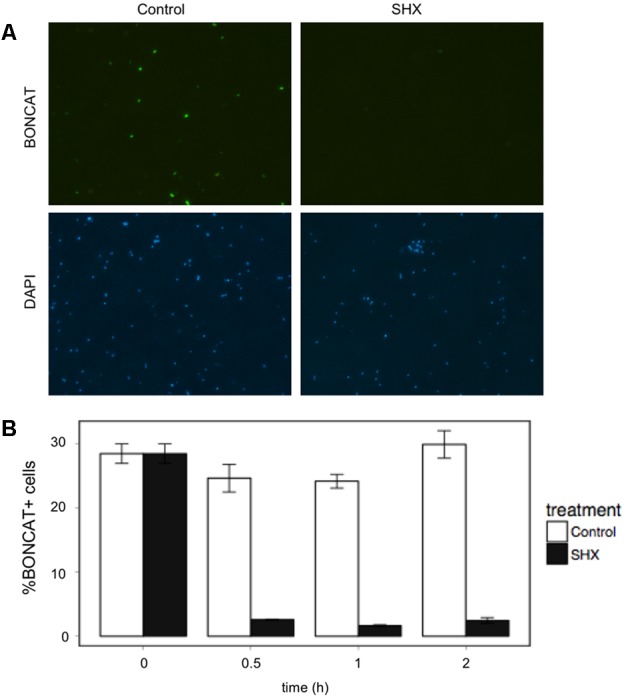
Bioorthogonal non-canonical amino acid tagging signal is not detected in cells treated with serine hydroxamate (SHX) which induces growth arrest. **(A)** Micrograph of BONCAT+ cells and DAPI-stained cells in a control treatment (left panel) and a SHX treatment. **(B)** Percentage of BONCAT+ cells in both treatments. SHX-treated cells displayed similar values to paraformaldehyde-killed controls. The fluorochrome CR110 azide was used for the BONCAT assay.

The amount of translationally active cells drastically decreased within 30 min of SHX addition (**Figure 5**), and remained within the range of the killed controls for the duration of the experiment (2 h), indicating that growth arrested cells do not display any BONCAT signal.

#### Changes in Activity during Manipulation Experiments

**FIGURE 6:**
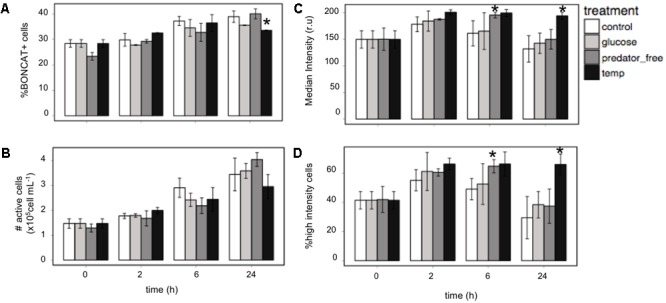
Visualizing activity changes in natural samples during a manipulation experiment with four different treatments: control, glucose addition, a treatment where predators were removed (see text for details), and a treatment where temperature was increased to 35°C, **(A)** percentage of BONCAT+ cells, **(B)** number of BONCAT+ cells, **(C)** median intensity of the fluorescence of BONCAT+ cells, and **(D)** percentage of high-fluorescence (top third of the fluorescence intensity) cells. Error bars represent the standard deviation of four replicates. Asterisks denote statistically significant differences from the control (mixed model, *p* < 0.05).

We further explored translational activity of natural samples from the Barcelona coast in manipulation experiments where water was subjected to different treatments: (i) one treatment in which predators had been removed, (ii) one treatment with glucose addition, and (iii) one treatment where temperature was increased up to 35°C. The percentage of BONCAT+ cells was similar in all treatments at the different time points, and only the temperature increase had a significant negative effect on the percent of BONCAT+ cells after 24 h (*p* < 0.05, mixed models, **Figure 6A**). When the number of active cells was considered instead of the percentage of active cells, no treatment produced a statistically significant effect, although removal of predators resulted in a slight increase in the number of active cells after 24 h (**Figure 6B**). In contrast, when the intensity of the fluorescence of the BONCAT signal was taken into account differences between treatments became more evident (**Figures 6C,D**). Temperature had a positive effect on the median intensity of the cells after 24 h of incubation, and also on the percentage of the cells with high fluorescence intensity (see section “Materials and Methods”) (**Figure 6D**). These results imply that, although the temperature treatment negatively affected the percentage of active cells, the cells that remained active had, on average, higher translational activity. Furthermore, removal of predators resulted in a significant increase in the more active cells (higher median intensity and larger percent of high fluorescence cells, **Figures 6C,D**) after 6 h of incubation. Taken together, these findings suggest that subpopulations within the community responded to temperature and the absence of predators at different time scales. Hence, BONCAT allows tracing changes in the single-cell activity of microbial assemblages.

### Optimizing the Substrate Concentration and Incubation Time for Oligotrophic Waters

**FIGURE 7:**
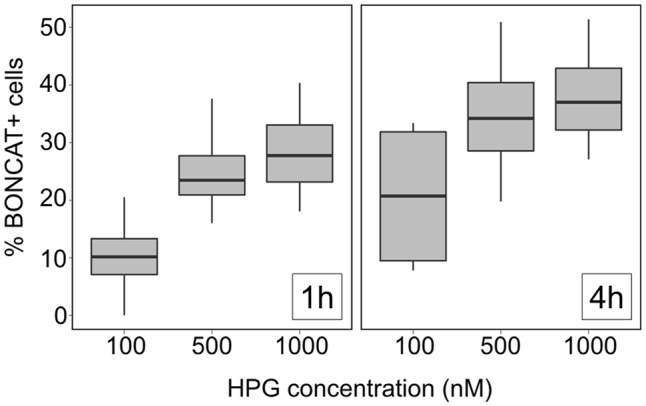
Effect of substrate (HPG) concentration and incubation time on the detection of BONCAT+ cells. Box plots represent the percentage of BONCAT+ cells in samples obtained at the Blanes Bay Microbial Observatory during 4 months (June to September 2016) using different HPG concentrations after 1 h (left panel) or 4 h (right panel) of incubation. The lower and upper hinges correspond to the first and third quartiles (the 25th and 75th percentiles), the solid line represents the median of the values, the whiskers represent 1.5 ^∗^ (the distance between the first and third quartiles).

The percentage of active cells generally increased at longer incubations times (**Figure 7**). However, there was a certain HPG concentration (500 nM, in our case) above which the number of active cells did not increase for a given incubation time. The fact that there is a saturation in the amount of cells detected above a given concentration threshold suggests high HPG does not result in an induction of inactive cells. Longer incubations times resulted in higher numbers of active cells, likely due to enhanced detection of slow-growing cells, as suggested in **Figure 4A**. We did not observe significant shifts in community composition in our samples within 4 h of incubation (data not shown), but long incubation times may lead to taxonomic changes. Therefore, there is a trade-off between detection and keeping the incubation time short enough so that community composition does not change, which should be ideally addressed for each particular system.

### Coupling BONCAT with CARDFISH

**FIGURE 8:**
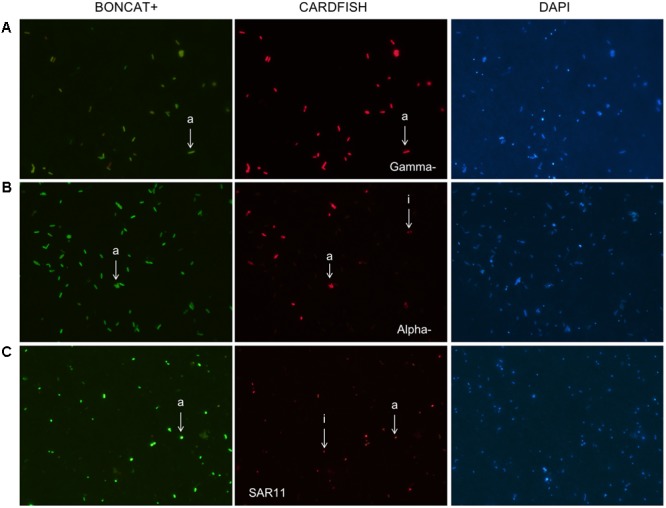
Coupling BONCAT with CARDFISH (CARDFISH–BONCAT) to link translational activity with microbial identity in natural samples. Micrographs of BONCAT (active cells), CARDFISH (probe+ cells), and DAPI-stained (total cells). **(A)** Gammaproteobacteria, **(B)** Alphaproteobacteria, and **(C)** SAR11 bacteria. Arrows indicate active (a) and inactive (i) cells. **(A,B)** Correspond to samples obtained during a mesocosm experiment. **(C)** Represents a sample taken from the beach of Barcelona.

Several combinations of fluorochromes were tested to establish the BONCAT–CARDFISH protocol, as detailed in Supplementary Table S1. In a published detailed-protocol for BONCAT (Hatzenpichler and Orphan, 2015), it is recommended to perform FISH after the click-reaction of BONCAT to minimize the potential for dissociation of the hybridized probes. This problem is avoided in CARDFISH due to the amplification step. By combining BONCAT and CARDFISH, we were able to identify active and inactive cells belonging to the Gammaproteobacteria, the Alphaproteobacteria, and SAR11 phylogenetic groups in natural samples (**Figure 8**). These results demonstrate the success of BONCAT–CARDFISH to identify bacterial cells that are active in the environment and their taxonomic affiliation.

## Discussion

Linking microbial diversity to ecosystem function is one of the major challenges in microbial ecology. With this goal in mind, there has been a considerable effort toward the development of single-cell techniques for addressing the activity and identity of microbes *in situ*, such as MARFISH, Raman-FISH, and HISH-SIMS (Alonso and Pernthaler, 2005; Huang et al., 2007; Musat et al., 2012). These techniques have produced valuable information about the role of bacteria in the environment, yet they all have considerable disadvantages as recently reviewed in Singer et al. (2017). Raman-FISH and HISH-SIMS have provided an unprecedented way of looking at microbial processes in the environment (Musat et al., 2012; Wang et al., 2016), but they rely on long incubations, rather expensive instrumentation and are relatively low throughput. MARFISH is the most widely used single-cell technique in the marine system and the use of radiolabeled substrates allows high sensitivity after short incubations, but sample processing takes days to weeks and requires a specialized laboratory. BONCAT has risen as an alternative technique to MARFISH, with a more simplified processing method that allows for faster results. In addition, it does not require a specialized facility and is relatively cost-effective if reagents are bought separately instead of purchasing ready-to-use kits (Hatzenpichler and Orphan, 2015). Hence, this technique can be easily implemented in any microbial ecology laboratory.

Although some BONCAT protocols were already available in the literature (Samo et al., 2014; Hatzenpichler and Orphan, 2015), we have optimized the protocol for its use with planktonic prokaryotes and in combination with CARDFISH. Attachment of the cells to the filters using low-gelling-point agarose (Pernthaler et al., 2002a), a permeabilization step (**Figure 2**), and the use of a coverslip during the click-reaction (**Figure 1**) have been implemented in our BONCAT protocol. The previous study with marine bacteria used a FTF technique in order to increase the signal-to-noise ratio (Samo et al., 2014), which may lead to unrealistic results, since some cells may remain attached to the filter. In contrast, the agarose treatment allows thorough washes to increase the signal-to-noise ratio with minimal cell loss. Permeabilization is crucial for CARDFISH, and although it is not needed for BONCAT detection of active cells, we observed that this treatment somehow enhanced the entry of the azide dye into the cell, and increased the signal-to-noise ratio, which may be important when working with low activity samples. The use of a coverslip during the click reaction also greatly enhanced the signal detection because it helped maintaining reducing conditions at the reaction site (**Figure 1**), as observed by Samo et al. (2014). We have now observed that the click-reaction can also be successfully performed in a completely filled Eppendorf tube (data not shown). In any case, both permeabilization and minimizing the exposure to oxygen should be implemented for low activity samples.

There is a strong heterogeneity in the growth rates (Ferrera et al., 2011) and the nutrients limiting different bacterioplankton groups (Sebastian et al., 2013). Thus, the incorporation of HPG may be variable depending on the taxonomic composition, the physiological state of the cells, and the environmental conditions. Samo et al. (2014) used only 20 nM of HPG for their experiments with water from the Scripps Pier (La Jolla, CA, United States). While this concentration worked for our experiments with water from the Barcelona beach and the bacterial cultures (**Figures 3, 6**), very few BONCAT+ cells were detected when using this concentration for the oligotrophic waters of the BBMO (data not shown). We found that higher concentration of HPG does not result in the induction of inactive cells (**Figure 7**), but longer incubation times seem to enable the detection of slow-growing bacteria or those with a slower metabolism. However, incubation times should be kept short enough to avoid changes in community composition (Hatzenpichler et al., 2016). Thus, ideally, HPG concentration and incubation time should be optimized for each system. If the tests necessary to find the optimum concentration and incubation time cannot be performed (for example, during an oceanographic cruise), based on our results, we recommend to use high concentrations of HPG (∼1–2 μM) and 2–3 h of incubation.

Overall, our results show that BONCAT provides a good estimation of the number of protein-synthesizing cells in a natural sample, and also enables monitoring the changes in activity of these cells as seen by changes in their fluorescence intensity (**Figures 3, 4, 6**). Samo et al. (2014) translated the BONCAT signal into protein synthesis rates using empirical conversion factors derived from experiments with isolates and natural samples. However, it has been argued that the fluorescence intensity may vary depending not only on the amount of proteins produced but also on the methionine content (because HPG is an analog for methionine) and the kinetics of incorporation of HPG, which may be different among phylotypes (Hatzenpichler and Orphan, 2015). Although we agree that quantifying the absolute amount of proteins synthesized is probably not feasible, we strongly believe that BONCAT coupled with CARDFISH constitutes a simple and powerful semi-quantitative approach to elucidate the relative contribution of different phylotypes to the bulk activity in a sample.

Bioorthogonal non-canonical amino acid tagging also allows proteomic analyses of newly synthesized proteins in response to a given environmental condition (Hatzenpichler et al., 2014), and it has been recently coupled with fluorescence activated cell sorting (FACS) to address which cells were active methane-oxidizers in deep sea methane seep sediments (Hatzenpichler et al., 2016). Thus, BONCAT in combination with all these techniques present great prospects for our understanding of microbial processes in the environment and the identity of the microbes involved.

## Author Contributions

MS planned and designed the experiments. AL, ME, and IF performed the analyses. AL and MS wrote the paper.

## Conflict of Interest Statement

The authors declare that the research was conducted in the absence of any commercial or financial relationships that could be construed as a potential conflict of interest.

## Footnotes

1 www.clickchemistrytools.com

2 www.clickchemistrytools.com

3 www.technobiology.ch
