# Supplementary material for: Using Click-Chemistry for Visualizing in Situ Changes of Translational Activity in Planktonic Marine Bacteria
Source: Front Microbiol. 2017 Dec 1;8:2360. doi: 10.3389/fmicb.2017.02360 (PMC5717025; doi:10.3389/fmicb.2017.02360)
Supplement: Supplementary file 1 [file Table_1.DOCX]

**Table S1**: Fluorochromes used in our tests for coupling BONCAT with CARDFISH

| **BONCAT fluorochrome**  **(azide form)** | **CARDFISH fluorochrome** | **Observations** |
| --- | --- | --- |
| **Cy3** | **Alexa 488** | There is an overlap in the emission spectra of both fluorochromes, so signals cannot be easily discriminated |
| **Alexa 594** | **Alexa 488** | Better signal-to-noise ratio if the click reaction is performed before CARDFISH |
| **Cr 110** | **Alexa 594** | Better signal-to-noise ratio if CARDFISH is performed before the click reaction |
